# Linear and Nonlinear Optical Properties of a Doubly Clamped Suspended Monolayer Graphene Nanoribbon Nanoresonator

**DOI:** 10.3390/mi13081179

**Published:** 2022-07-26

**Authors:** Spyridon G. Kosionis, Emmanuel Paspalakis

**Affiliations:** Materials Science Department, School of Natural Sciences, University of Patras, 265 04 Patras, Greece; kosionis@upatras.gr

**Keywords:** exciton, phonon, linear susceptibility, graphene nanoribbon nanoresonator, third-order susceptibility

## Abstract

We studied the optical properties of a hybrid structure that was composed of a semiconductor quantum dot and a doubly clamped suspended graphene nanoribbon nanoresonator. We obtained analytical results for the linear and third-order optical susceptibilities of the hybrid system. The spectrum of the linear susceptibility exhibited a single resonance, and its position depended on the value of the on-resonance exciton energy and the exciton–nanoribbon resonator coupling strength coefficient; the amplitude of the resonance was independent of the values of these parameters. The third-order optical susceptibility spectrum exhibited a sharp resonance arising at low frequencies of the probe field, the position of which depended only on the frequency of the fundamental flexural phonon mode. It also presented a broader resonance arising at higher frequencies of the probe field, the position of which was determined both by the coupling strength coefficient and by the exciton frequency; its amplitude depended solely on the exciton–photon coupling strength.

## 1. Introduction

The optical response of hybrid systems that make use of the interaction between different excitations, such as, for example, excitons from semiconductor quantum dots or molecules and plasmons from metallic nanoparticles, have attracted the interest of recent studies due to the enhanced nonlinear and quantum optical effects that the hybrid systems may offer, as compared to their uncoupled constituents [1,2,3,4,5,6,7,8,9,10,11,12,13,14,15,16,17,18,19]. Another important category of hybrid systems investigated for their enhanced nonlinear optical response are those emerging due to the coupling between excitons in quantum dots and phonons in nanoresonators [20,21,22,23,24,25,26,27,28,29]. In these latter studies, the quantum system was described as a two-level system interacting with a weak probe field that was also strongly pumped by a coherent coupling field. The coupling of the quantum system, which provides the exciton, with the nanoresonator, which provides a system of low mass and high vibrational frequency, contributes importantly to all the possible optical effects that arise due to the exciton–phonon coupling. These unique characteristics make such hybrid systems ideal for applications in sensing with extreme sensitivity, ultrafast optical switching, and efficient optical storage. 

The type of the nanoresonator that was used differed in the various studies; the quantum system was coupled to a suspended Z-shaped graphene nanoribbon [20,21,22], a carbon nanotube [23,24,25], a monolayer MoS2 suspended on a Si/SiO_2_ substrate [26], or DNA molecules [27,28,29]. In these hybrid systems, the main nonlinear optical effects that have already been studied are the linear dispersion/absorption under coherent pump–probe excitation and the cross-Kerr nonlinearity under the action of a strong pump field. The nonlinear optical response of the system is determined by the intensity and the frequency of the pump field and the exciton–phonon coupling. In the hybrid systems with quantum dots or molecules and metallic nanostructures, it was shown that even in the absence of the pump field, the optical response of the hybrid structure could lead to several interesting effects that depended on the exciton–plasmon coupling [10,11,12,17]. Interestingly, to the best of our knowledge, the linear and nonlinear optical properties of an exciton from a quantum system coupled to a nanoresonator in the absence of the pump field has not been explored so far. This was the purpose of the present publication.

Specifically, we analyzed the optical properties of a hybrid structure that was composed of a quantum dot and a doubly clamped suspended graphene nanoribbon nanoresonator, similar to that studied in [20,21,22]. Graphene-based nanomechanical resonators have been studied for over 15 years and have very interesting properties [30,31,32]. In addition, it has been shown that a quantum dot could be trapped in a Z-shaped graphene nanoribbon junction [33,34]. The combination of these two structures provided the hybrid structure that we studied in this work when it interacted with a weak probe laser field. We first derived the Langevin equations of motion and obtained analytical results for the linear and third-order optical susceptibilities of the system. The spectrum of the linear susceptibility exhibited a single resonance, the position of which was dependent both on the value of the on-resonance exciton energy and on the exciton–nanoribbon resonator coupling strength coefficient. However, the width and the amplitude of the resonances were independent of these parameters. The third-order optical susceptibility spectrum exhibited a sharp resonance arising at low frequencies of the probe field, the position of which depended exclusively on the frequency of the fundamental flexural phonon mode and a broader resonance arising at higher frequencies of the probe field; its position was determined both by the coupling strength coefficient and by the exciton frequency, and its amplitude was solely dependent on the exciton–phonon coupling strength. 

## 2. Theory

**Figure 1:**
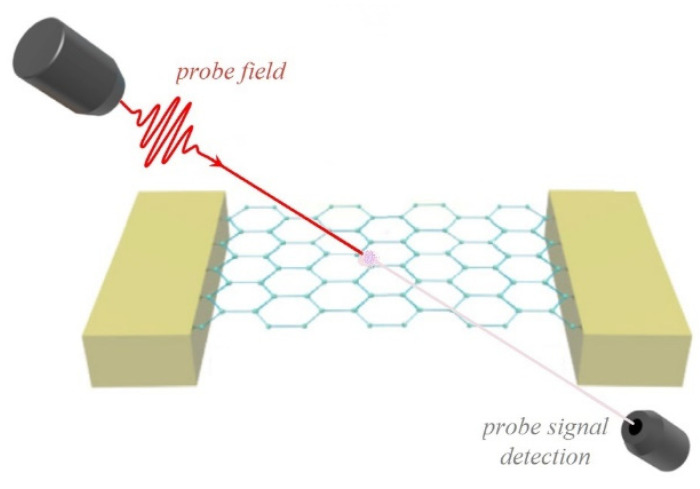
Schematic representation a doubly clamped suspended monolayer Z-shaped graphene nanoribbon nanoresonator coupled to an exciton while interacting with a probe electromagnetic field.

**Figure 2:**
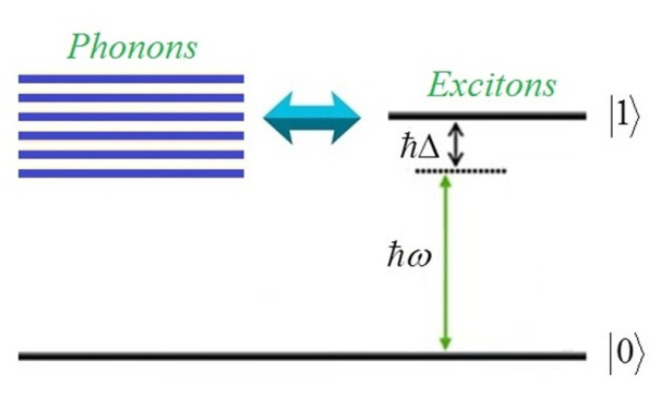
The energy level scheme of the hybrid system. The continuum of the energy states represents the phonon states, while the ground state $|0\rangle$ and the excited state $|1\rangle$ describe the two-level excitonic system.

The hybrid system under study was a doubly clamped suspended graphene nanoribbon nanoresonator that was coupled to a small-scale localized exciton formed in its vicinity (Figure 1). We assumed that the quantum system interacted with an electromagnetic field of amplitude E and angular frequency ω. The ground and the excited states of the localized exciton are respectively denoted as $|0\rangle$ and $|1\rangle$. The energy level scheme of the hybrid system is depicted in Figure 2, which shows an exciton being created due to the excitation of the two-level system, while a continuum of the energy states represents the phonon states of the graphene nanoresonator. 

In this study, we aimed at the derivation of analytical expressions for the linear susceptibility $\chi^{\left(1\right)}$ and the nonlinear susceptibility $\chi^{\left(3\right)}$ in a systematic and straightforward manner. In order to perform the calculation of these physical quantities, we followed the methodology presented below, starting with the Hamiltonian that describes the system in a rotating frame with respect to the probe field’s angular frequency:

(1) $$H=\hbar \Delta \sigma_{z}+\hbar \omega_{n}a^{+}a+\hbar g\sigma_{z}(a+a^{+})-\hbar (\Omega e^{-i\omega t}+\Omega^{*}e^{i\omega t})(\sigma_{10}+\sigma_{01}).$$

Here, $\hbar \omega_{n}a^{+}a$ is the vibration Hamiltonian term of the graphene resonator, where $\hbar \omega_{n}$ is the fundamental phonon mode energy that is consistent with the exciton transition, since the vibration modes can be treated as phonon modes. The creation and the annihilation operators for the eigenmode of the graphene are respectively denoted as $a^{+}$ and a. The exciton was coupled to the phonon mode of the graphene nanoresonator; we symbolized the strength of this coupling with the parameter g. The pseudospin operators are represented by $\sigma_{z}$, $\sigma_{01}$, and $\sigma_{10}$. Moreover, $\Delta =\omega_{10}-\omega$ is the detuning of the probe field from the $|1\rangle ↔|0\rangle$ resonance, Ω=μE/ℏ is the Rabi frequency, μ denotes the dipole moment element corresponding to the transition, and $\hbar \omega_{01}=({Ε}_{1}-{Ε}_{0})$ expresses the energy of the exitonic transition. The relaxation rate of the atomic coherence associated with the density matrix element $\rho_{nm}$ and the population decay rate corresponding to the transition $|n\rangle ↔|m\rangle$ (n≠m) are indicated by $\gamma_{nm}$ and $\Gamma_{nm}$, respectively. 

If we substitute the expression of the Hamiltonian of Equation (1) into the Heisenberg equation, we derive the following Langevin equations of motion:

(2) $$\dot{p}(t)=-\Gamma_{2}p(t)-i\omega_{10}p(t)-igp(t)\Xi (t)-i\Omega e^{-i\omega t}w(t)+i\Omega^{*}e^{i\omega t}w(t),$$

(3) $$\dot{w}(t)=-\Gamma_{1}-\Gamma_{1}w(t)+2i(\Omega^{*}e^{i\omega t}+\Omega e^{-i\omega t})[p^{*}(t)-p(t)],$$

(4) $$\ddot{\Xi }(t)+\gamma_{n}\dot{\Xi }(t)+\omega_{n}^{2}\Xi (t)=-\omega_{n}gw(t).$$

In Equations (2)–(4), we introduce the operator notation $p=\langle \sigma_{01}\rangle$, $w=2\langle \sigma_{z}\rangle$, and $\Xi =\langle a+a^{+}\rangle$. In order to examine the linear and the third-order optical response of the system, we first calculated the corresponding terms that are introduced in the expression of the optical susceptibility of the system. Thus, we proceed to the third-order expansion of the density matrix elements, with respect to the Rabi frequency of the probe field, as follows:

(5) $$\begin{array}{l} K_{i}=K_{i}^{\left(1\right)}+\Omega^{*}e^{i\omega t}K_{i}^{\left(2\right)}+\Omega_{}e^{-i\omega t}K_{i}^{\left(3\right)}+{\left(\Omega^{*}\right)}^{2}e^{2i\omega t}K_{i}^{\left(4\right)}+{\left|\Omega \right|}^{2}K_{i}^{\left(5\right)}+\Omega^{2}e^{-2i\omega t}K_{i}^{\left(6\right)}\\ {{}_{}}_{}{{}_{}}_{}{{}_{}}_{}{}_{}+{\left(\Omega^{*}\right)}^{3}e^{3i\omega t}K_{i}^{\left(7\right)}+\Omega^{*}{\left|\Omega \right|}^{2}e^{i\omega t}K_{i}^{\left(8\right)}+\Omega {\left|\Omega \right|}^{2}e^{-i\omega t}K_{i}^{\left(9\right)}+\Omega^{3}e^{-3i\omega t}K_{i}^{\left(10\right)} \end{array}$$

with $K_{1}=p$, $K_{2}=w$, and $K_{3}=\Xi$; and $|K_{i}^{\left(1\right)}|>>|K_{i}^{\left(2\right)}|,|K_{i}^{\left(3\right)}|>>|K_{i}^{\left(4\right)}|,$$\left|K_{i}^{\left(5\right)}|,|K_{i}^{\left(6\right)}|,|K_{i}^{\left(7\right)}|,|K_{i}^{\left(8\right)}|,|K_{i}^{\left(9\right)}|,|K_{i}^{\left(10\right)}\right|$. After introducing these expressions in Equations (2)–(4), we derive a set of 30 differential equations. The susceptibility of the system is defined as:

(6) $$\chi =\frac{(\Gamma /V)\mu p}{\varepsilon_{0}E_{0}}$$

and can be analyzed in a Taylor series. If we maintain terms up to the third order in our expansion, we take:

(7) $$\chi =\chi^{\left(1\right)}+3\chi^{\left(3\right)}E_{0}^{2},\;\mathrm{with}$$

(8) $$\chi^{\left(1\right)}=\frac{\mu^{2}(\Gamma /V)}{\varepsilon_{0}\hbar \Omega }A_{3}^{*}\;\mathrm{and}$$

(9) $$\chi^{\left(3\right)}=\frac{\mu^{4}(\Gamma /V)}{3\varepsilon_{0}\hbar^{3}\Omega^{3}}A_{9}^{*},$$

respectively representing the linear and the third-order susceptibility, where $\varepsilon_{0}$ is the dielectric constant of vacuum, while V and Γ represent the volume of the semiconductor quantum dot and the optical confinement factor related to the exciton transition, respectively. If we also define the following simplified notation for the coefficients defined in Equation (5):

(10) $$K_{1}^{\left(n\right)}=A_{n},\;K_{2}^{\left(n\right)}=B_{n},\;K_{3}^{\left(n\right)}=C_{n}$$

and we assume that the Rabi frequency is a real parameter, analytical expressions can be derived for the coefficients $A_{3}^{*}$ and $A_{9}^{*}$ in a steady state:

(11) $$\begin{array}{ll} A_{3}^{*} & =-\frac{\Omega }{\left(\omega -\omega_{10}-g^{2}/\omega_{n}\right)-i\Gamma_{2}}\\ & =\frac{\Omega \left[\omega -\left(\omega_{10}+g^{2}/\omega_{n}\right)\right]}{{\left[\omega -\left(\omega_{10}+g^{2}/\omega_{n}\right)\right]}^{2}+{\left[\left(2\Gamma_{2}\right)/2\right]}^{2}}+i\frac{\left(\Omega /\Gamma_{2}\right){\left[\left(2\Gamma_{2}\right)/2\right]}^{2}}{{\left[\omega -\left(\omega_{10}+g^{2}/\omega_{n}\right)\right]}^{2}+{\left[\left(2\Gamma_{2}\right)/2\right]}^{2}} \end{array}$$

and

(12) $$A_{9}^{*}=\frac{\left[g(A_{2}^{*}C_{6}^{*}+A_{3}^{*}C_{5}^{*})+\Omega (B_{5}^{*}-B_{4})\right]}{\left(\omega -\omega_{10}-g^{2}/\omega_{n}\right)-i\Gamma_{2}},$$

where

(13) $$A_{2}^{*}=-\frac{\Omega }{\left(\omega +\omega_{10}+g^{2}/\omega_{n}\right)+i\Gamma_{2}},$$

(14) $$B_{4}=\frac{2\Omega (A_{3}^{*}-A_{2})}{2\omega -i\Gamma_{1}},$$

(15) $$B_{5}^{*}=\frac{2i\Omega }{\Gamma_{1}}(A_{2}^{*}-A_{2}+A_{3}^{*}-A_{3}),$$

(16) $$C_{5}^{*}=-\frac{2i\Omega g}{\omega_{n}\Gamma_{1}}(A_{2}^{*}-A_{2}+A_{3}^{*}-A_{3})$$

and

(17) $$C_{6}^{*}=\frac{\omega_{n}g\Omega (A_{2}-A_{3}^{*})}{\left\{2\left[\omega^{2}-{\left(\omega_{n}/2\right)}^{2}\right]-i\omega \gamma_{n}\right\}(2\omega -i\Gamma_{1})}.$$

At this point, the analytical expressions of the coefficients $A_{3}^{*}$ and $A_{9}^{*}$ given by Equations (11) and (12) can be introduced into Equations (8) and (9) to calculate the linear and the third-order optical susceptibility, respectively. 

## 3. Results

**Figure 3:**
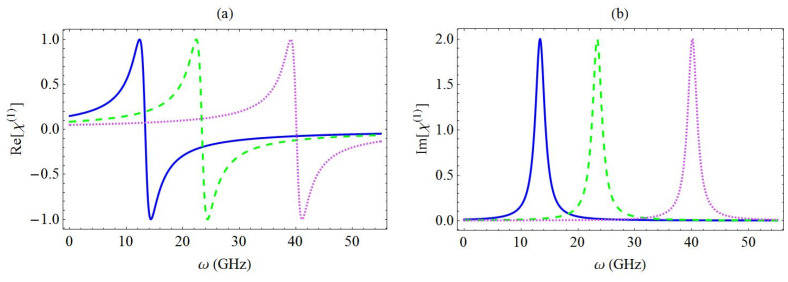
Spectra of the real part (**a**) and the imaginary part (**b**) of $\chi^{\left(1\right)}$ for different values of the coupling strength between the exciton and the phonon mode: g=5 GHz (blue solid curve), 10 GHz (green dashed curve), and 15 GHz (magenta dotted curve). In addition, $\omega_{10}=10\;GHz$.

**Figure 4:**
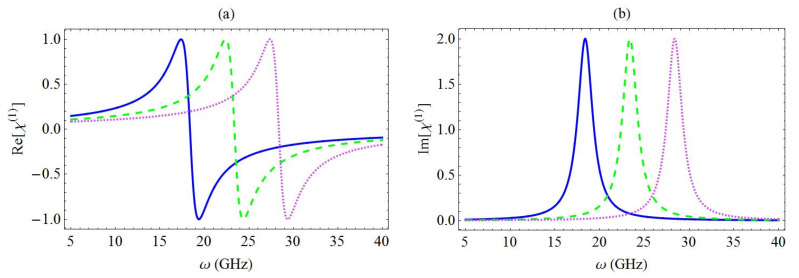
Spectra of the real part (**a**) and the imaginary part (**b**) of $\chi^{\left(1\right)}$ for different values of the exciton energy: $\omega_{10}=5\;GHz$ (blue solid curve), 10 GHz (green dashed curve), and 15 GHz (magenta dotted curve). In addition, g=10 GHz.

In all the figures presented below, the frequency of the fundamental flexural phonon mode $\omega_{n}$ was taken equal to 7.477 GHz, and the decay rate of the nanoresonator was $\gamma_{n}=\omega_{n}/Q$, with Q=9000, which is considered to be a comparatively high quality factor [20,21]. The exciton decay and dephasing rates were $\Gamma_{1}=2_{}GHz$ and $\Gamma_{2}=1_{}GHz$, respectively, as in [20]. In Figure 3 and Figure 4, the response of the $\chi^{\left(1\right)}$ spectrum as a function of the probe field angular frequency was investigated, as the coupling strength parameter was modified. More specifically, in Figure 3, we depicted the spectra of the real part (a) and the imaginary part (b) of $\chi^{\left(1\right)}$, as the coupling strength between the exciton and the phonon mode varied. For simplicity, in Figure 3, we exhibited the spectrum of the dimensionless coefficient $A_{3}^{*}$. The blue solid curve corresponds to g=5 GHz, while the green dashed curve and the magenta dotted curve are for the cases of g=10 GHz and g=15 GHz, respectively. The required exciton angular frequency $\omega_{10}$ was assumed to be equal to $10_{}\;GHz$. When we introduced the expression of Equation (11) in Equation (8), it became evident that we should expect the linear optical susceptibility spectra to exhibit a single resonance when the value of the probe field angular frequency ω was exactly equal to $\omega_{10}+g^{2}/\omega_{n}$. This means that the resonances observed in Figure 3, arising at the positions with ω=13.3 GHz, 23.4 GHz, and 40.1 GHz, which were respectively taken for $g=5_{}\;GHz$, $10_{}\;GHz$, and $15_{}\;GHz$, were transposed to the right at an accelerating rate, as the coupling strength increased monotonically. More specifically, as was theoretically predicted, the transposition of the resonances was analogous to the second power of the coupling coefficient. Based on the analytical expression of $A_{3}^{*}$ (Equation (11)), we deduced that the imaginary part of $\chi^{\left(1\right)}$ (Equation (8)) corresponded to a Lorentzian-shaped profile with amplitude $\mu^{2}(\Gamma /V)/\left(\varepsilon_{0}\hbar \Gamma_{2}\right)$ and a full width at half maximum equal to $2\Gamma_{2}$. We noted that these quantities were both independent of the coupling strength coefficient g and the exciton resonance frequency $\omega_{10}$. Furthermore, the real part of $\chi^{\left(1\right)}$ had the characteristic dispersion-like profile near resonance, the amplitude and the width of which were also independent of the parameters g and $\omega_{10}$, and are respectively given by the analytical expressions $\mu^{2}(\Gamma /V)/\left(2\varepsilon_{0}\hbar \Gamma_{2}\right)$ and $2\Gamma_{2}$. 

In Figure 4, the spectra associated with the real part (a) and the imaginary part (b) of the linear optical susceptibility are presented for different values of the exciton energy. Here, the blue solid curve, the green dashed curve, and the magenta dotted curve correspond to $\omega_{10}=5_{}\;GHz$, $10_{}\;GHz$, and $15_{}\;GHz$, respectively, for a specific value of the coupling coefficient (g=10 GHz). As mentioned above, the resonance was theoretically predicted to arise at the angular frequency $\omega =\omega_{10}+g^{2}/\omega_{n}$ and, hence, the transposition of the resonance along the horizontal axis exhibited a linear dependence on $\omega_{10}$. We noted that the position of the resonances was also dependent on the value of the resonator’s fundamental frequency $\omega_{n}$. However, in the present study, this parameter was assumed to be constant (equal to $7.477_{}\;GHz$), as mentioned above. 

**Figure 5:**
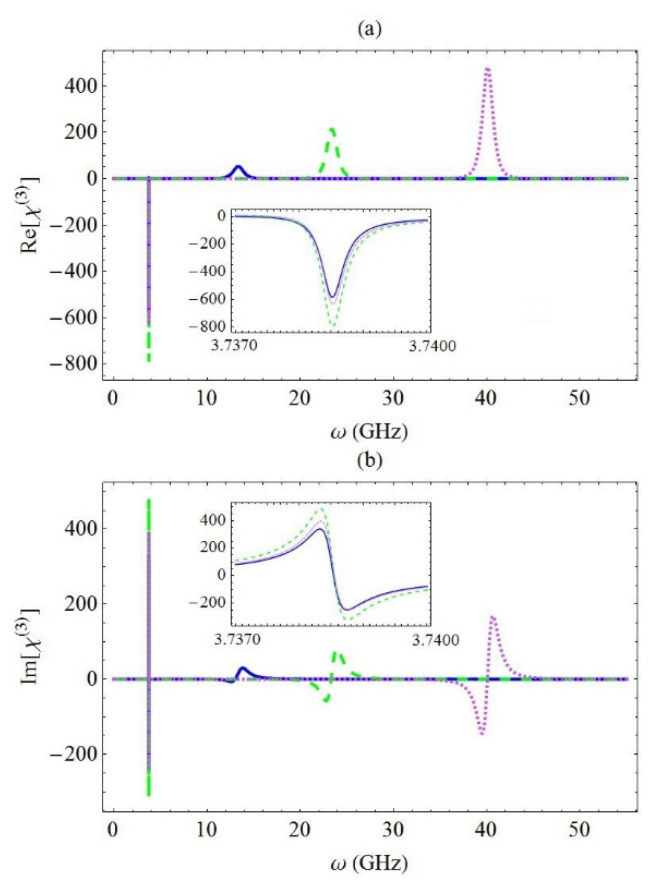
The real part (**a**) and the imaginary part (**b**) of $\chi^{\left(3\right)}$, for different values of the coupling strength between the exciton and the phonon mode: g=5 GHz (blue solid curve), 10 GHz (green dashed curve), and 15 GHz (magenta dotted curve). In addition, $\omega_{10}=10\;GHz$.

In Figure 5, we present the spectra of the real part (a) and the imaginary part (b) of the third-order optical susceptibility $\chi^{\left(3\right)}$ for the same values of the exciton–phonon mode coupling strength as the ones considered in Figure 3, in which the spectra of the linear optical susceptibility were explored. For simplicity, in Figure 5, we present the spectra of the real and the imaginary part of the dimensionless coefficient $A_{9}^{*}$. If we substituted Equation (12) into Equation (9), we noted that the spectral profile of $\chi^{\left(3\right)}$ should exhibit a sharp resonance centered around $\omega =\omega_{n}/2$ (which was found by setting the denominator of the $C_{6}^{*}$ coefficient equal to zero) and a broader resonance placed at $\omega =\omega_{10}+g^{2}/\omega_{n}$ (which could also be derived based on the mathematical formula of the $A_{9}^{*}$ coefficient). These resonances, both being theoretically predicted, were indeed observed in the spectra presented in Figure 5. We noted that the left resonance, which arose on the spectrum of Re[χ(3)] at $\omega =\omega_{n}/2$, was quite sharp, and it also presented an inverse Lorentzian profile, while the resonance detected in Im[χ(3)] exhibited a Fano-type profile. These resonances were observed in the insets inserted in captions (a) and (b), respectively. We noted that the amplitude and the position of the resonance, which were centered around $\omega =\omega_{10}+g^{2}/\omega_{n}$, were both proportional to the square of the coupling strength coefficient between the exciton and the phonon mode, as was theoretically predicted. On the other hand, the amplitude of the resonance detected at $\omega =\omega_{n}/2$ did not really seem to exhibit a well-specified pattern, as the values of the physical parameters of the system were modified. However, the position of this resonance was totally unaffected by the modification of the coupling strength between the nanoresonator and the exciton, as expected.

**Figure 6:**
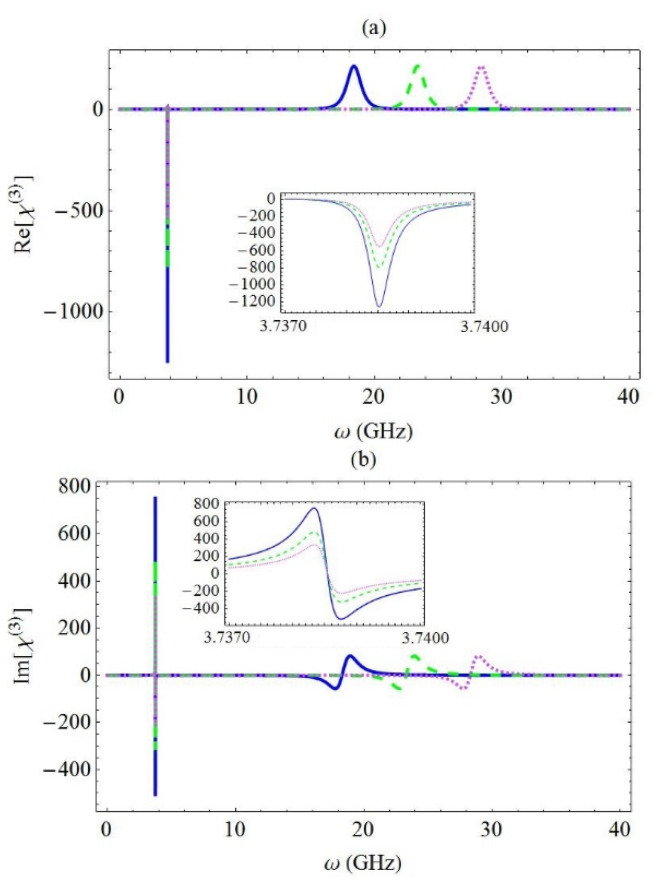
The real part (**a**) and the imaginary part (**b**) of $\chi^{\left(3\right)}$, for different values of the exciton energy: $\omega_{10}=5\;GHz$ (blue solid curve), 10 GHz (green dashed curve), and 15 GHz (magenta dotted curve). In addition, g=10 GHz.

Finally, in Figure 6, the real part (a) and the imaginary part (b) of the third-order optical susceptibility $\chi^{\left(3\right)}$ are presented for different values of the exciton resonance frequency. More specifically, the blue solid curve corresponds to $\omega_{10}=5\;GHz$, while the green dashed curve and the magenta dotted curve correspond to 10 GHz and 15 GHz, respectively. In addition, the exciton–phonon coupling strength g was equal to 10 GHz, as in the case of the $\chi^{\left(1\right)}$ spectra presented in Figure 4. Since the value of the fundamental phonon mode $\omega_{n}$ was not modified, the position of this resonance did not practically change. However, the resonance arising at $\omega =\omega_{10}+g^{2}/\omega_{n}$ was transposed to the right when the exciton resonance frequency is increased, since its position presented linear dependence on the $\omega_{10}$ parameter. Hence, equally spaced values of the exciton energy led to a set of equidistant spectral peaks. We also observed that when $\omega_{10}$ increased, the amplitude of the sharp resonance decreased at a decelerating rate, whereas the amplitude of the broader resonance remained intact. 

## 4. Conclusions

In this work, we examined the linear and third-order optical responses of a doubly clamped suspended graphene nanoribbon nanoresonator that was coupled to a small-scale localized exciton. We assumed that the entire system interacted with a probe electromagnetic field, while the fundamental phonon mode of the nanoresonator was consistent with the exciton transition of a two-level system. The Hamiltonian was introduced in the Heisenberg equation, and the relative Langevin equations of motion were derived. We next applied a third-order Taylor expansion of the density matrix elements with respect to the Rabi frequency of the probe field and obtained analytical expressions for the linear and the third-order optical susceptibility of the system. The spectrum of the linear optical susceptibility presented a single resonance that had a typical near-resonance dispersion profile in the spectrum of its real part, and also exhibited a single peak in the spectrum of its imaginary part. The position of the resonance presented a linear dependence on the second power of the strength characterizing the coupling between the exciton and the phonon mode, as well as on the exciton resonance frequency, whereas its width and its amplitude were independent of the value of these parameters. In the third-order spectral response, we observed a sharp dip (Fano-type resonance) on the spectrum of its real/imaginary resonance arising at low values of the frequency of the probe field, as well as a broader Lorentzian peak (Fano-profile resonance) arising at higher values of the frequency of the probe field. The position of the sharp resonance depended solely on the frequency of the fundamental flexural phonon mode, whereas the position of the broader resonance exhibited the same patterns as the ones discovered for the resonance observed in the profile of the linear susceptibility. The amplitude of the broad resonance was proportional to the square of the coupling strength coefficient, whereas it did not depend on the characteristic value of the exciton resonance frequency. The dependence of the characteristics associated with the resonances in the exciton–phonon coupling constituted a strong indication that this effect may be useful in sensing applications. 

## Data Availability

The data presented in this study are available on request from the corresponding author.
